# Association Between Gout and All-Cause as well as Cardiovascular Mortality: A Systematic Review

**DOI:** 10.1007/s11926-011-0234-2

**Published:** 2012-02-18

**Authors:** Kathrin Lottmann, Xiaoyu Chen, Peter K. Schädlich

**Affiliations:** IGES Institut GmbH, Friedrichstraβe 180, 10117 Berlin, Germany

**Keywords:** Gout, Mortality, All-cause mortality, Cardiovascular mortality, Systematic review, Association, Crystal arthritis

## Abstract

Gout affects 1% to 2% of the population, and the prevalence is increasing due to changes in diet and the ageing of the population. Its development and risk factors have been explored frequently, and recommendations for the diagnosis and management of gout implemented. Nevertheless, there is a lack of knowledge regarding the long-term impact on gouty patients. This systematic review therefore evaluates the association between gout and all-cause as well as cardiovascular mortality. A systematic literature search was performed, and seven long-term studies were ultimately analyzed. Six of them used multivariate regressions to assess the adjusted mortality ratio in gouty patients with reference to patients without the disorder. Despite differences in study designs, study populations, and definitions of gout, the results were consistent: There was an independent association between gout and all-cause as well as cardiovascular mortality. Knowing that patients with gout are at risk emphasizes the need for adequate care.

## Introduction

Gout is a common disease with increasing prevalence. Its manifestation, progression, adequate therapeutic interventions, and related comorbidities are therefore frequently discussed in the literature. Less attention is paid to the question of whether gout has an impact on mortality. However, this long-term outcome is relevant not only for the patient. New light should also be shed on the significance of this disease and consequently on the treatment and care of gouty patients, as these often remain suboptimal [1].

Gout is a crystal deposition disease in which renal elimination of uric acid is insufficient. The increased uric acid level can lead to the formation of monosodium urate crystals in synovial fluid or soft tissues. This results in painful inflammation of the joints, which is why the disorder is also known as *inflammatory arthritis* [1, 2]. The typical initial presentation of gout is podagra [3]. Gout is characterized by recurrent acute episodes and can become a chronic condition [3–5].

The prevalence of gout has increased in recent decades. This might be the result of a changing diet and an ageing population [2, 3]. Despite methodologic challenges, the international prevalence is estimated at up to 1% to 2% [1, 6, 7]. Men are more likely to be affected than women, and the prevalence increases with age [1, 8].

In 2006, the European League Against Rheumatism (EULAR) developed evidence-based recommendations for the diagnosis and management of gout [1, 9], according to which the disease may be diagnosed by monosodium urate crystals in synovial fluid, podagra, or tophus, while a raised serum urate level alone is not specific to gout [1]. Risk factors for the development of gout are diet [2, 5]; genetic predisposition [2, 4]; hyperuricemia [4, 10]; and comorbidities such as diabetes, hypertension, obesity, heart failure, and renal insufficiency [2, 6, 11].

The management of gout aims at lowering and maintaining serum urate levels below the saturation point (6.8 mg/dL, or 408 μmol/L). This helps to dissolve existing monosodium urate crystals and to prevent further crystals from forming [1, 9]. In addition, special diets, weight reduction, and reduced alcohol consumption are among the nonpharmacologic interventions [4].

The objective of this review is to scrutinize in a systematic manner whether there is an association between gout and all-cause or cardiovascular mortality. Despite neither the management of gout nor economic aspects being considered, this systematic review is intended to impact on recurrent discussion about the management of gout. Furthermore, knowledge about the association of gout and mortality may underline the need for adequate care.

## Methods

### Data Sources

We conducted a systematic literature search of Medline und EMBASE up to April 2011 using a search strategy combining the Medical Subject Headings (MeSH terms) and keywords in the titles/abstracts. The search string consisted of keywords referring to the medical indication gout linked by the Boolean operator “AND” to terms associated with the outcomes of all-cause mortality or cardiovascular mortality. Publications in English, French, and German were included, whereas studies on animals were excluded. In addition, we performed a manual search of references.

### Study Selection

Studies were included in this review if they were noninterventional trials investigating the association between gout and all-cause or cardiovascular mortality in patients with gout compared with the population without this disease. Gout could be defined by the respective *ICD* codes (*ICD-9* code 274.x or *ICD-10* code M10.x [12, 13]) or diagnosed by a physician in accordance with the evidence-based EULAR recommendations [1]. The patient-relevant outcomes considered were all-cause mortality and cardiovascular mortality (*ICD-9* code 390–459, *ICD-10* code I00–I99) [12, 13]. Studies analyzing the association between a single cardiovascular disease (CVD) (eg, the number of fatal myocardial infarctions) and gout were therefore excluded because this systematic review focuses on cardiovascular mortality as a whole. In addition, studies of the best available and most feasible evidence were to be included. With regard to the question posed by this review, the best evidence would be a retrospective cohort study with a 2b level of evidence [14].

### Validity Assessment and Data Abstraction

Two investigators (Lottmann and Chen) independently scrutinized all identified studies after excluding duplicates. As a first step, the titles and abstracts were investigated with regard to the predefined inclusion criteria, and the full texts of all potentially relevant studies were evaluated next. In case of disagreement, consensus was reached by discussion. The selection process, including the reasons for exclusion, was documented. Due to the lack of a validated quality assessment tool for nonrandomized interventions [15], we developed our own checklist [16]. Our quality checklist was based on the methodologic requirements of the German Agency for Health Technology Assessment [17] and on the Strengthening the Reporting of Observational Studies in Epidemiology (STROBE) guidelines [18]. Finally, the quality of each study included was assessed and scored. The data from all studies scored with at least “fair” methodologic quality were subsequently collected by one reviewer using a self-developed data abstraction list.

## Results

### Study Identification and Selection

**Fig. 1:**
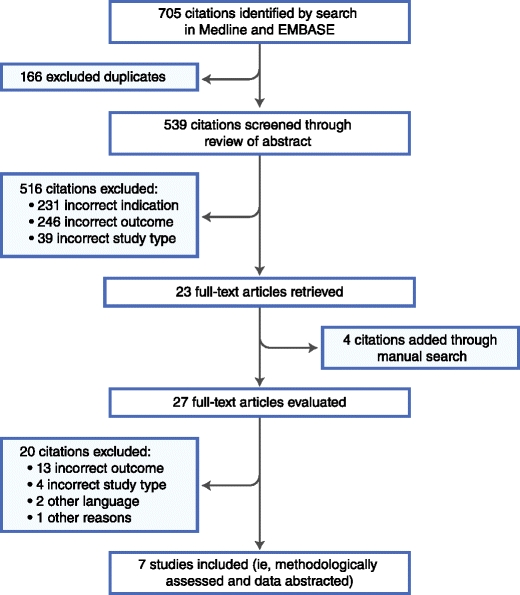
Flow diagram of study identification and selection. (*Adapted from* Khan et al. [16])

The process of the systematic literature search is depicted in Fig. 1. The number of articles identified, screened, and excluded, as well as the number of full-texts retrieved and finally included for data abstraction is delineated. We identified seven articles as conforming to our inclusion criteria. All of them analyzed the association between gout and all-cause mortality (seven studies) as well as cardiovascular mortality (four studies) as either a primary or secondary outcome.

The study types varied from retrospective to prospective. They consisted of cohort studies, surveys, and data analyses. All these studies, except one [19], analyzed the association between gout and mortality using multivariate regression (ie, they calculated hazard ratios [HRs] or risk ratios [RRs]). In addition, several studies determined the mortality rate in patients with gout compared with people without this disease. In this review, we focus on the results of the regression analyses, as this is the more appropriate way of obtaining conclusive results.

### Patient Characteristics

Patient populations were very heterogeneous in the different studies, which made comparison challenging. Whereas two studies included renal transplant populations [20, 21], another two focused on individuals with coronary heart disease (CHD) or those who were at risk of this disorder [22••, 23]. One study analyzed data from US veterans [19], while another included only data from health professionals [24]. An additional study evaluating participants in a health-screening program in Taiwan was included. Even if an almost equal sex ratio in the study population was provided, gout was far more common in men than in women (90.4% of the patients with gout were male) [25••]. Furthermore, several studies were conducted in an older population. Despite this heterogeneity, each study population was characteristic of gouty patients. Study populations showed typical comorbidities, age groups, and male dominance in patients with gout.

The definition of gouty patients varied from study to study, as did the prevalence of gout in the study population (range, 2.1%–10.6%). The 1-year mortality rate from all causes also varied (range, 2.6%–14.3%).

### Gout and All-Cause Mortality

Abbott et al. [20] used Medicare claims data of primary renal transplant patients reported in the United States Renal Data System (USRDS). The aim of their retrospective cohort study was to evaluate the implications of new-onset gout on survival. They determined an increased risk of mortality (adjusted HR [AHR], 1.26 [95% CI, 1.08–1.47]) in patients developing gout after kidney transplantation compared with renal transplant patients without the disorder. Gout diagnosed prior to transplant was not significantly associated with an increased risk of mortality (AHR, 1.18 [95% CI, 0.98–1.43]). Their study indicates an independent association between new-onset gout after renal transplantation and all-cause mortality.

One more study evaluated the association between incident gout in renal transplant patients and mortality using the USRDS. This study population was treated with dialysis after the transplantation, and gout was associated with an increased risk of mortality (AHR, 1.49 [95% CI, 1.43–1.55]; *P* < 0.001) [21]. The result not only confirmed the independent association with new-onset gout after renal transplantation but even determined an elevated mortality risk in kidney transplant patients treated with dialysis [21] compared with those without dialysis [20].

The association between gout and all-cause mortality in patients with cardiovascular comorbidities was investigated in a time-matched, nested case-control analysis of a retrospective cohort study based on the Quebec Universal Health Insurance database. The study population (age >66 years) had been discharged from the hospital with a primary diagnosis of heart failure. Death was considered to be associated with gout if the patient suffered an acute episode of gout within 60 days before death. Regarding the gout population, a distinction was made between three definitions of gout. The risk of all-cause mortality in patients with any acute gout was significantly increased, even if adjusted for several CVDs and medications (adjusted RR [ARR], 1.76 [95% CI, 1.08–2.86]; *P* = 0.02). No significant association between acute gout and all-cause mortality was found in patients admitted to the hospital for gout. However, in patients visiting an emergency department for a gout flare, gout was again significantly associated with an increased risk of death from all causes (ARR, 2.10 [95% CI, 1.19–3.70]; *P* = 0.01) [22••].

Whether there is an association between gout and all-cause mortality in men at above average risk of CHD but without evidence of clinical CVD during the intervention phase was evaluated in the long-term follow-up of the MRFIT (Multiple Risk Factor Intervention Trial) study over 17 years. Again, different definitions of gout were used: self-reported gout and documented hyperuricemia, self-reported gout alone, and prescribed gout medication. Gouty patients based on self-reported gout and documented hyperuricemia had an increased mortality risk (AHR, 1.22; *P* = 0.009) compared with those patients with neither gout nor hyperuricemia. The mortality risk in gouty patients, defined by use of gout-related medicine (allopurinol, probenecid, or colchicine), was comparable (AHR, 1.23; *P* < 0.001) [23]. The association between gout and all-cause mortality was revealed once again, although hyperuricemia might have biased the result in one of the analyses.

A further study focused on patients with gout and cardiovascular comorbidities [24]. In the prospective Health Professionals Follow-Up Study, which was limited to male participants, data were acquired via mailed questionnaires. The authors calculated the adjusted mortality risk in patients with gout and with CHD, as well as in gouty patients without CHD comorbidity (in each case compared with patients without gout but with the corresponding CHD status). They furthermore evaluated different definitions of gout: any gout, history of gout, and newly diagnosed gout. Patients with a history of gout without CHD had a slightly higher mortality risk (ARR, 1.28 [95% CI, 1.15–1.41]) than those with a history of gout and CHD comorbidity (ARR, 1.25 [95% CI, 1.09–1.45]). Moreover, an increased mortality risk was determined in patients with newly diagnosed gout (ARR, 1.28 [95% CI, 1.13–1.46]); CHD comorbidities were not reported. In the analysis including any gouty patients, the mortality risk was slightly lower in the group without CHD (ARR, 1.25 [95% CI, 1.13–1.38]) than in the group with CHD (ARR, 1.35 [95% CI, 1.21–1.50]) [24]. This study thus reported an association between gout and all-cause mortality independent of the CHD status and not affected by the gout duration.

While all the studies mentioned above included mainly Caucasians as well as a few black patients, the association between gout and all-cause mortality was also confirmed in an Asian population. Data from a health-screening program in Taiwan were investigated. This analysis revealed an increased risk of death from all causes in patients with gout (AHR, 1.46 [95% CI, 1.12–1.91]; *P* = 0.005) [25••]. The mortality risk was even higher when a more verifiable definition of gout was used and the analysis included only patients with monosodium urate crystals in the synovial fluid or *ICD* code 274.x, and excluded those with self-reported gout (AHR, 1.51 [95% CI, 1.14–1.99]; *P* = 0.004).

One study did not calculate an RR to evaluate the association between gout and all-cause mortality but accounted for a multivariable-adjusted difference in the mortality rate. Based on a mailed survey to US veterans (99% men), similar mortality rates were determined in individuals with and without gout (no gout, 2.22% [95% CI, 1.56%–3.15%]; gout, 2.62% [95% CI, 1.60%–4.28%]; difference, 18.47%; *P* = 0.230) [19]. Gout therefore did not have an impact on the mortality rate in this study. However, conclusions regarding the association between gout and all-cause mortality should be drawn carefully, as the statistical methods used differ from those in the other studies described previously. Detailed figures are displayed in Table 1.

**Table 1:** Overview of study characteristics and results on the association between gout and all-cause/cardiovascular mortality

Study (year)	Duration, *y*	Population	All-cause mortality: AHR or ARR (95% CI)	Cardiovascular mortality: AHR or ARR (95% CI)
Abbott et al. [20] (2005)	≤3	28,924 patients with renal transplantation	1.26 (1.08–1.47)	NA
		• 1,583 new-onset gout after transplantation	1.18 (0.98–1.43)	
		• 1,175 new-onset gout prior to transplantation		
Choi and Curhan [24] (2007)	12	51,297 men (health professionals)	No CHD, 1.25 (1.13–1.38)	No CHD, 1.32 (1.09–1.60)
		• 2,773 with gout	CHD, 1.35 (1.21–1.50)	CHD, 1.35 (1.19–1.55)
Cohen et al. [21] (2008)	≤6	259,209 dialysis patients with renal transplantation (48.4% men)	1.49 (1.43–1.55); *P* < 0.001	1.49 (1.43–1.55); *P* < 0.001
		• 24,215 with gout (39.9% men)		
Krishnan et al. [23] (2008)	17	9,105 men at risk of CHD		
		• 655 self-reported gout and hyperuricemia	Self-reported gout and hyperuricemia, 1.22; *P* = 0.009	Self-reported gout and hyperuricemia, 1.30 (1.04–1.61); *P* = 0.02
		• 974 gout-related medicine	Use of gout-related medicine, 1.23; *P* < 0.001	Use of gout-related medicine, 1.18; *P* = 0.08
Kuo et al. [25••] (2010)	≤8	61,527 participants in a Taiwanese health-screening program	1.46 (1.12–1.91); *P* = 0.005	1.97 (1.08–3.59); *P* = 0.027
		• 1,311 with gout (90.4% men)		
Thanassoulis et al. [22••] (2010)	≤8	25,090 patients aged >66 y discharged from hospital with primary diagnosis of heart failure	1.76 (1.08–2.86); *P* < 0.02	NA
		• 1,053 with gout		
Singh and Strand [19] (2008)	1	64,553 US veterans	NA	NA
		• 1,581 with gout (99% men)	Adjusted 1-y mortality rate: 2.63% with gout, 2.22% without gout; difference, 18.47%; *P* = 0.230	

AHR, adjusted hazard ratio; ARR, adjusted risk ratio; CHD, coronary heart disease; CI, confidence interval; NA, not applicable

### Gout and Cardiovascular Mortality

Besides the described independent association between gout and all-cause mortality, four of the seven identified studies evaluated the impact of gout on cardiovascular mortality as well. All of them revealed that gout is also associated with an increased risk of cardiovascular mortality; the results are outlined in detail in Table 1. The cardiovascular mortality risk in gouty renal transplant patients treated with dialysis was increased compared with those patients without gout (AHR, 1.47 [95% CI, 1.26–1.59]). However, this mortality ratio was slightly lower than the all-cause mortality risk in the same group [21].

Even in the long-term follow-up study over 17 years, gout was associated with cardiovascular mortality, but the risk changed when data were limited to special definitions of gout. Based on self-reported gout and diagnosed hyperuricemia, the cardiovascular mortality risk was only significantly higher when the reference group consisted of patients with neither gout nor hyperuricemia (AHR, 1.30; *P* = 0.02). The risk was not significantly increased when compared with patients without gout but potentially with hyperuricemia. Two sensitivity analyses with gouty patients defined as 1) using gout medication and 2) having self-reported gout revealed a nonsignificantly increased risk of cardiovascular mortality [23].

The independent association between gout and cardiovascular mortality was also reported in the Health Professionals Follow-Up Study. In this case, the cardiovascular mortality risk in patients with gout was even slightly higher than the all-cause mortality risk for the same group. Again, the analysis of risk was carried out for different gout groups, depending on the duration of illness (history of gout, newly diagnosed gout, and any gout) and on the CHD status (history of gout [no CHD]: ARR, 1.38 [95% CI, 1.15–1.66]; history of gout [CHD]: ARR, 1.26 [95% CI, 1.07–1.50]; newly diagnosed gout [CHD not reported]: ARR, 1.31 [95% CI, 1.08–1,59]; any gout [no CHD]: ARR, 1.32 [95% CI, 1.09–1.60]; any gout [CHD]: ARR, 1.35 [95% CI, 1.19–1.55]) [24].

Compared with the previously reported study results, the cardiovascular mortality risk determined in the study with participants in the Taiwanese health-screening program was higher. The main analysis including gouty patients corresponding to at least one of the three applied gout definitions of this study revealed an almost twofold higher cardiovascular mortality risk in patients with gout compared with those without the disease (AHR, 1.97 [95% CI, 1.08–3.59]; *P* = 0.027). When data were limited to patients with monosodium urate crystals in the synovial fluid or corresponding to *ICD* code 274.x, and excluded those with self-reported gout, the risk was somewhat lower (AHR, 1.86 [95% CI, 1.01–3.44]; *P* = 0.048) [25••].

### All-Cause and Cardiovascular Mortality Risks in Gouty Subgroups

Different subgroup analyses revealed an independent association between gout and all-cause mortality as well as cardiovascular mortality not only in the respective study populations as a whole, but also in several subgroups. Results for single subgroups are presented accounting for the impact of comorbidities on the mortality risk.

#### Sex

Male gouty patients after renal transplantation who were treated with dialysis had a lower risk of all-cause mortality than women with the same characteristics (AHR, 1.59 [95% CI, 1.47–1.71] vs AHR, 1.64 [95% CI, 1.54–1.75]) [21].

#### Age

The Health Professionals Follow-Up Study revealed an increased all-cause mortality risk in the population 60 to 69 years of age compared with patients without gout from the same age group. This mortality risk decreased as patients grew older. In the youngest age group (<60 years), the increased mortality risk was not significant (<60 years: AHR, 1.30 [95% CI, 0.96–1.74]; 60–69 years: AHR, 1.35 [95% CI, 1.13–1.61]; ≥70 years: AHR, 1.16 [95% CI, 1.01–1.32]). The cardiovascular mortality risk in patients with gout in the 60- to 69-year subgroup was more than two times higher than in patients without gout from the same age group; the risks in the other age groups were not significantly increased (<60 years: AHR, 1.84 [95% CI, 0.93–3.61]; 60–69 years: AHR, 2.10 [95% CI, 1.38–3.18]; ≥70 years: AHR, 0.98 [95% CI, 0.68–1.41]) [24]. Thus, gouty patients 60 to 69 years of age seem to be at elevated all-cause and cardiovascular mortality risk.

#### Race

The AHR for all-cause mortality was higher in black patients with gout than in other races (AHR, 1.69 [95% CI, 1.59–1.85] vs AHR, 1.59 [95% CI, 1.50–1.69]) [21].

#### Gout Duration

Newly diagnosed gout seems to have a greater impact on all-cause mortality than established gout (≤5 years: AHR, 1.44; 6–10 years: AHR, 1.37; 11–15 years: AHR, 1.30; >15 years: AHR, 1.32) [24].

#### Hypertension

The mortality risk in gouty patients in the Health Professionals Follow-Up Study both with and without hypertension was almost the same (ARR, 1.24 [95% CI, 1.09–1.41] vs ARR, 1.25 [95% CI, 1.06–1.47]), while subgroup analysis for cardiovascular mortality did not reveal a significant AHR in patients with gout [24]. By contrast, dialysis-treated gouty renal transplant patients with hypertension had a higher mortality risk than those without hypertension (AHR, 1.74 [95% CI, 1.55–1.94] vs AHR, 1.59 [95% CI, 1.51–1.68]) [21].

#### Hypercholesterolemia

Men with gout and hypercholesterolemia have a lower all-cause mortality risk than those without this comorbidity (with hypercholesterolemia: ARR, 1.15 [95% CI, 0.96–1.37] vs without hypercholesterolemia: ARR, 1.30 [95% CI, 1.15–1.47]) [20]. The influence of hypercholesterolemia did not change with reference to cardiovascular mortality—gouty patients without hypercholesterolemia seem to have a higher mortality risk compared with those with hypercholesterolemia (with hypercholesterolemia: ARR, 1.31 [95% CI, 0.93–1.83] vs without hypercholesterolemia: ARR, 1.55 [95% CI, 1.05–2.30]). Not all results were significant [24].

#### Diabetes

Diabetes in patients with gout appears to decrease all-cause mortality slightly, as the adjusted risk was lower than in gouty patients without diabetes (AHR, 1.56 [95% CI, 1.44–1.69] vs AHR, 1.68 [95% CI, 1.58–1.78]) [21].

## Discussion

Despite the differences among populations in the studies included, all multivariate regressions yielded the same result: There was an independent association between gout and all-cause mortality as well as cardiovascular mortality. Conclusions regarding the causes of this association cannot be drawn, as further research would be needed. This also applies to the question of whether there is an association between cardiovascular mortality and all-cause mortality. However, gout elevates the risk of mortality and thus makes gouty patients an at-risk population.

Patients in whom gout was newly diagnosed after a renal transplantation had an increased all-cause mortality risk, whereas this association was not significant in renal transplant patients with a history of gout [20]. Another study confirmed the impact of gout on all-cause as well as cardiovascular mortality in renal transplant patients treated with dialysis [21]. Furthermore, an independent association between gout and all-cause mortality as well as cardiovascular mortality was also found in patients with any gout or a history of this disorder, irrespective of CHD comorbidities [24]. Another study confirmed the association between gout and all-cause mortality in patients with previous heart failure and acute gout within 60 days before their death [20]. Gouty patients with hyperuricemia also had an increased all-cause and cardiovascular mortality risk [23]. Finally, a study of an Asian population without specific comorbidities obtained the same result (ie, it indicated that gout is associated with all-cause as well as cardiovascular mortality) [25••].

The number of studies evaluating the association between gout and all-cause or cardiovascular mortality is limited. More research has been conducted on the questions regarding whether gout is associated with death from CHD alone or CVD in general, and the impact of gout on cardiovascular events [26–29]. Even these studies indicate corresponding results: Gout is a risk factor for CVD and for death caused by it.

The large number of study participants in each of the studies included strengthens the validity of the results, although the prevalence of gout in the study population was somewhat higher than the general estimated prevalence [2, 6, 7]. Despite the heterogeneous study populations and differences in gout definition, all studies indicated an independent association between gout and all-cause as well as cardiovascular mortality (except for the study that calculated differences in the mortality rates instead of ARR/AHR) [19]). That all multivariate regressions yielded consistent outcomes may be considered another validation of gout being a risk factor for mortality. Moreover, the specific characteristics of the study population reflected the typical characteristics of gouty patients: mainly male, older age, and comorbidities such as CVD or renal failure.

In spite of the consistent outcomes, the comparability of the studies is complicated by use of different definitions of gout. Wijands et al. [30] described the challenge of defining gout in epidemiologic studies. It is difficult to apply diagnostic guidelines (eg, monosodium urate crystals), which are developed for use on an individual level, to large populations. Drawing on *ICD* codes might offer a solution but implies several methodologic uncertainties [30]. As a consequence, different definitions of a gout population might entail variant outcomes. This is in line with the studies analyzed in this review: The mortality risk varied depending on the gout definition [22••, 23, 25••]. To resolve this challenge, several sensitivity analyses were conducted.

The impact of patient characteristics and comorbidities on the association between gout and mortality was determined in different subgroup analyses. Regarding race, subgroup analysis established a higher mortality risk in blacks [21], which goes hand-in-hand with an increased risk of gout among this ethnic group. Contrary to the elevated risk of gout in men described in the literature, one study indicated a reverse effect, as the risk of all-cause mortality was higher in women than men [21]. It could be concluded that gout is less common, but more serious, in women. Furthermore, results of a subgroup analysis with diabetes, one of the most common comorbidities in gout, were unexpected. Gouty patients with diabetes had a slightly lower mortality risk than those without this comorbidity [21]. One explanation might be that monitoring is better in patients with diabetes, although further research is needed to verify this claim. These results should be interpreted with caution, as they originate from a study of patients with severe comorbidities (renal transplant patients treated with dialysis).

The effect of gout duration on the mortality risk was analyzed in only two studies, both of which reported an increased mortality risk in patients with new-onset gout and a slightly lower risk over time [20, 24]. This indicates the need for early intervention and treatment of gout.

Finally, it should be noted that four of the seven studies included were explicitly not sponsored by pharmaceutical companies [19, 21, 22••, 25••], two were partially supported by the industry [23, 24], and information on sponsorship or conflict of interest was lacking in one study [20].

## Conclusions

Gout is independently associated with all-cause mortality and cardiovascular mortality. This was the message of six studies evaluating the association by multivariate regression. As described, study populations were heterogeneous, but the study outcomes were nevertheless consistent. The elevated mortality risk in patients with gout applies to gouty patients with typical characteristics (black, male, older age) and classical comorbidities (eg, diabetes, hypertension, hyperuricemia). Subgroup analyses suggest that patients with new-onset gout and female sex have a slightly elevated mortality risk. In addition to these two subgroups, gouty patients without comorbidities that require regular monitoring, such as diabetes, might be considered a vulnerable group. Not only these subgroups, but the generally increased mortality risk associated with gout should be borne in mind when treating this disorder. A need exists for adequate treatment of gout to fulfill the needs of these at-risk patients.
